# ENVIRONMENTALLY SUSTAINABLE DIET AND RECYCLING THERAPY FOR OLDER ADULTS’ WELL-BEING AND HEALTHCARE EXPENDITURES

**DOI:** 10.1093/geroni/igz038.2689

**Published:** 2019-11-08

**Authors:** Hsinyi Hsiao, Aaron Hagedorn

**Affiliations:** 1 Tzu Chi University, Hualien, Taiwan, Taiwan; 2 University of Southern California, Los Angeles, California, United States

## Abstract

Due to the rapid climate change in recent years, global communities have seen an increased frequency of large-scale natural and man-made disasters. Acknowledging extravagant lifestyles and wasteful consumption of natural resources as prominent factors leading to intensified stress placed on the biosphere and ecosystems, many older adults with chronic medical conditions or disabilities in Taiwan have promoted environmental sustainability through two ways: 1) participating a recycling program in their local communities by collecting, sorting, and reclaiming reusable resources that are regenerated into eco-friendly blankets for disaster survivors and 2) practicing plant-based diet to promote healthy lifestyle and mitigate climate change. These older participants of environmentally sustainable initiatives believe the interconnectedness among humanity, the environment, and sustainable lifestyles. To understand association between the recycling therapy and older adults’ well-being a quasi-experimental design with 72 people age 60 and older (recycling group vs. non-recycling group) and photovoice were used to examine longitudinal effects of the recycling therapy on physical and mental health. To examine the effects of plant-based diet on dementia and health care cost ten-year data from Taiwan’s National Health Insurance Database were used by two studies to compare risk of dementia and medical expenditure between vegetarian and non-vegetarian groups among older adults in Taiwan. Four presentations in the symposium will discuss positive outcomes of environmental volunteering on older adults’ physical and mental wellness and longitudinal effects of a cohort with environmental-friendly diet on dementia risk and lower actual health care expenditures.

